# Distribution, characterization, and induction of CD8^+ ^regulatory T cells and IL-17-producing CD8^+ ^T cells in nasopharyngeal carcinoma

**DOI:** 10.1186/1479-5876-9-189

**Published:** 2011-11-04

**Authors:** Jiang Li, Zhou-Feng Huang, Geng Xiong, Hao-Yuan Mo, Fang Qiu, Hai-Qiang Mai, Qiu-Yan Chen, Jia He, Shu-peng Chen, Li-Min Zheng, Chao-Nan Qian, Yi-Xin Zeng

**Affiliations:** 1 State Key Laboratory of Oncology in South China, Sun Yat-Sen University Cancer Center, Guangzhou, China; 2 Department of Nasopharyngeal Carcinoma, Sun Yat-Sen University Cancer Center, Guangzhou, China; 3 Department of Biotherapy, Sun Yat-Sen University Cancer Center, Guangzhou, China

**Keywords:** Nasopharyngeal carcinoma, Tumor-infiltrating lymphocytes, CD8^+ ^regulatory T cells (Tcreg), IL-17-producing CD8^+ ^T cells (Tc17)

## Abstract

**Background:**

CD8^+ ^effector cells often have an antitumor function in patients with cancer. However, CD8^+^Foxp3^+ ^regulatory T cells (Tcregs) and interleukin (IL)-17-producing CD8^+ ^T cells (Tc17 cells) also derive from the CD8^+ ^T cell lineage. Their role in the antitumor response remains largely unknown. In the present study, we aimed to investigate the distribution, characterization, and generation of CD8^+ ^Tcregs and Tc17 cells in NPC patients.

**Methods:**

Peripheral blood and tumor biopsy tissues from 21 newly diagnosed patients with nasopharyngeal carcinoma (NPC) were collected, along with peripheral blood from 21 healthy donors. The biological characteristics of Tcregs and Tc17 cells from blood and tumor tissues were examined by intracellular staining, tetramer staining and fluorescence-activated cell sorting (FACS) analysis. The suppressive function of Tcregs was investigated using a proliferation assay that involved co-culture of sorted CD8^+^CD25^+ ^T cells with naïve CD4^+ ^T cells *in vitro*.

**Results:**

We observed an increased prevalence of Tcregs and Tc17 cells among tumor-infiltrating lymphocytes (TILs) and different distribution among peripheral blood mononuclear cells (PBMCs) in NPC patients. Cytokine profiles showed that the Tcregs expressed a high level of IL-10 and low level of transforming growth factor β, whereas Tc17 cells expressed a high level of tumor necrosis factor α. Interestingly, both subsets expressed a high level of interferon γ in TILs, and the Tcregs suppressed naïve CD4^+ ^T cell proliferation by a cell contact-dependent mechanism *in vitro*. Moreover, we demonstrated the existence of Epstein-Barr virus latent membrane protein (LMP) 1 and LMP2 antigen-specific Tcregs in NPC.

**Conclusions:**

Our data provide new insights into the composition and function of CD8^+ ^T-cell subsets in NPC, which may have an important influence on NPC immunotherapy.

## Background

Undifferentiated nasopharyngeal carcinoma (NPC) is associated with Epstein-Barr virus (EBV) infection, which has a high incidence in Southern China and Southeast Asia. Conventional therapy is often ineffective for NPC patients with late-stage disease [1,2]. Recently, immunotherapy has become a promising therapeutic option for various types of cancer with high immunogenicity, including NPC [3-5]. The success of EBV-specific cytotoxic T lymphocyte (CTL) treatment has been reported in NPC. However, it has been difficult to obtain consistent results or stable clinical efficacy [6-8]. One possibility is that immune-suppressive environments may be created in patients with NPC [9-11]. Thus, a better understanding of the immune status of NPC patients, including the distribution of specific lymphocyte subsets and their functions, is crucial in developing more effective immunotherapy strategies.

CD8^+ ^T cells that express high levels of the transcription factors Eomes and T-bet are usually destined to develop into cytotoxic effector cells that produce interferon (IFN) γ, granzyme B and perforin, however, CD8^+ ^cells may also give rise to a regulatory lineage (Tcreg). The CD4^+^Foxp3^+ ^regulatory T cells (Tregs), which have been recognized as a suppressor of antitumor immunity because of its natural suppressive effect on the proliferation and IL-2 secretion of naïve and effector T cells [12,13], but the distribution, generation, characteristics, and function of Tcregs in cancer remain poorly understood, as does their pathogenic antigen specificity. Furthermore, interleukin (IL)-17-producing CD8^+ ^T cells (Tc17 cells) have been identified in mice and humans, and their enrichment inside solid tumors has been reported [14]. However, the generation and function of Tc17 cells in cancer remain largely uncharacterized.

In this study, we aimed to investigate the distribution, characterization, and generation of CD8^+ ^Tcregs and Tc17 cells in NPC patients. We observed an increase of Tcregs and decrease of Tc17 cells from peripheral blood mononuclear cells (PBMCs) and an accumulation of Tcregs and Tc17 cells in tumor tissues from 21 NPC patients. The Tcreg subset expressed CC chemokine receptor 6 (CCR6), cytotoxic T lymphocyte antigen 4 (CTLA4), and glucocorticoid-induced tumor necrosis factor (TNF)-related (GITR) proteins at high levels, resulting in a Treg-like phenotype. The Tc17 cells expressed high levels of CCR6 and low levels of CTLA4 and GITR protein, and they contained a high proportion of cells secreting TNFα, which is a Th1 cytokine. Moreover, the Tcregs from the tumor-infiltrating lymphocytes (TILs) secreted high levels of IL-10 and IFNγ but low levels of IL-2, IL-4, TNFα, and IL-17, resulting in a Tr1-like cytokine profile; Tcregs from PBMCs, in contrast, secreted high levels of IL-10 and low levels of transforming growth factor β (TGFβ), IL-2, IFNγ, TNFα, and IL-17. In addition, there was a significantly higher percentage of IFNγ-secreting cells among the Tc17 cells from the TILs than among those from the PBMCs, and this was true of cells from both NPC patients and healthy donors. Furthermore, the Tcregs from NPC patients possessed a suppressive function to the proliferation of CD4^+ ^naive T cells, which was mainly mediated by a cell-to-cell contact-dependent mechanism. IFNγ failed to impair the suppressive function of Tcregs *in vitro*. Collectively, these data suggest that, in addition to CD8^+ ^cytolytic effector cells, Tcregs and Tc17 cells with diverse functions are present in NPC patients. Additionally, this is the first demonstration of the existence of EBV antigen-induced Tcregs in patients with NPC.

## Materials and methods

### Samples

Tumor biopsy tissues and blood samples were collected from 21 newly diagnosed patients with NPC in Sun Yat-Sen University Cancer Center between 2009 and 2010. The tissue samples were cut into pieces. In addition to pathological diagnosis, one portion was used to generate and expand TILs, and another portion was used for fluorescence-activated sorting (FACS) analysis. Prior to FACS analysis, cells were briefly cultured in low dose IL-2 (20 IU/ml) RPMI 1640 complete medium to obtain a sufficient number of lymphocytes. PBMCs were isolated from the blood samples of 21 patients with NPC and 21 age-matched healthy donors; the samples had been frozen for FACS analysis. This study was conducted in accordance with the Helsinki Declaration, with written informed consent provided by the patients and with approval from the Research Ethics Committee of the Sun Yat-Sen University Cancer Center. Written consent was also obtained from all healthy donors before their participation.

### Generation of TILs and FACS analysis

Bulk TILs were isolated from the NPC biopsy specimens by mincing the tissue into small pieces and digesting with collagenase type IV. Cells were then cultured in RPMI 1640 medium that contained 10% human AB serum supplemented with L-glutamine, 2-mercaptoethanol, and recombinant human IL-2 (300 IU/ml) so as to generate T cells, as described previously [15].

The expression of markers on T cells was investigated by FACS analysis after surface or intracellular staining with specific antibodies that were conjugated to different fluorescent dyes (eBioscience, San Diego, CA, USA). Intracellular staining for IL-17 and other cytokines was performed on T cells stimulated by phorbol12-myristate13-acetate (PMA) and ionomycin for 4 h in the presence of brefeldin A (10 μg/ml, Sigma-Aldrich). Intracellular cytokines and Foxp3 were detected using a fixation and permeabilization protocol and buffers that were purchased from eBioscience.

The proportion of T cells that were specific for HLA-A2-restricted epitopes in LMP1 and LMP2 were analyzed by staining with HLA-A2 tetramers. The tetramers were assembled with synthetic peptides that originated from LMP1 (YLQQNWWTL) and LMP2 (LLWTLVVLL and GLGTLGAAI) (Guangzhou Taimo Corporation, Guangzhou, China). Aliquots of 0.5-1 × 10^6 ^cells were incubated at 4°C for 30 min in PBS that contained 1% fetal calf serum and 1 μg/ml phycoerythrin (PE)-labeled tetrameric complex. The samples were stained with anti-CD8-FITC and anti-Foxp3-APC antibodies and were then fixed in 0.5% paraformaldehyde for 20 min. For each sample, 10^5 ^cells were analyzed using an FC500 flow cytometer and CXP analysis software (Beckman Coulter, Inc., Fullerton, CA, USA).

### Sorting and expansion of CD8^+^CD25^+ ^Tcregs and suppression assay

CD8^+^CD25^+ ^Treg cells from PBMCs or TILs were stained with anti-CD8 and anti-CD25 antibodies and were sorted by FACS (MoFlo XDP Cell Sorter; Beckman Coulter, Inc.). Proliferation assays were performed as described previously [16]. In brief, 10^5 ^CD4^+ ^naïve T cells or CD8^+ ^effector T cells were labeled with 5- or 6-(N-Succinimidyloxycarbonyl)-3', 6'-O, O'-diacetylfluorescein (CSFE, Invitrogen) and co-cultured with CD8^+ ^Tregs at the indicated ratios. Cells were co-cultured for 5 days in 96-well round-bottom plates coated with human anti-CD3 (1 μg/ml), with or without the presence of anti-IL-10 or anti-IFNγ antibodies. The proliferation of CSFE-labeled CD4^+ ^naïve T cells was detected by FACS analysis. Transwell experiments were performed in 24-well plates using inserts with a pore size of 0.4 μm (Corning Gilbert Inc, Arizona, USA).

### Statistical analysis

Numerical data were expressed as means ± standard error. Statistical differences between the means for the different groups were evaluated with SSPS 13.0 software using a one-way analysis of variance with the level of significance at *P *< 0.05.

## Results

### Distribution and proportion of CD8^+ ^Tcregs and Tc17 cells in patients with NPC

**Figure 1:**
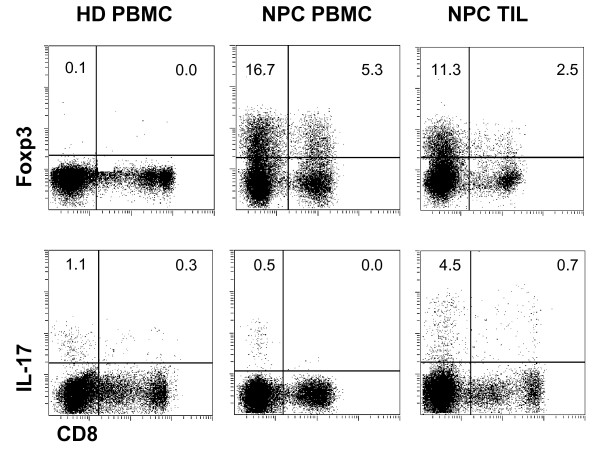
**Representative FACS dot plot for CD8 and Foxp3 staining or CD8 and IL-17 staining of PBMCs from healthy donors or PBMCs and TILs from one paired NPC patient**.

**Figure 2:**
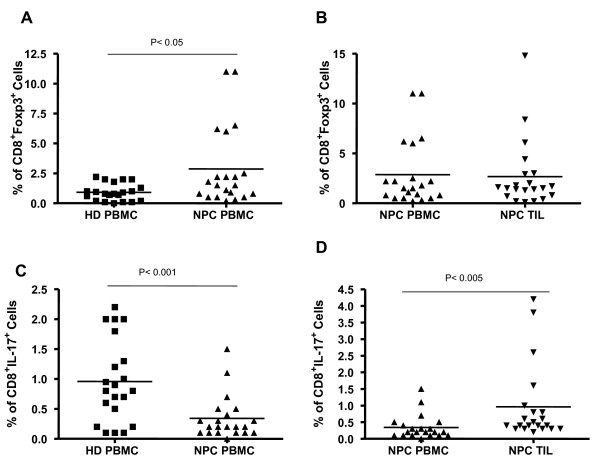
**Distribution of Tcregs and Tc17 cells in PBMCs and TILs from paired NPC patients**. The proportion of Tcregs (A, B) and Tc17 cells (C, D) in total CD8^+ ^T cells from PBMCs and freshly isolated TILs of paired NPC patients (*n *= 21) and healthy donors (HD, *n *= 21). Subsets were identified by intracellular staining of Foxp3 and IL-17 after PMA/ionomycin stimulation *in vitro*. Cumulative data show a significant increase in the number of Tcregs in PBMCs and TILs of NPC patients as compared with PBMCs of healthy donors and a significant decrease in Tc17 cells from PBMCs of NPC patients as compared with TILs from NPC patients and PBMCs from healthy donors.

Our previous study showed that the density of CD8^+ ^TILs is not a favorable prognostic factor for NPC patients [17]. Here, we continued to investigate the characteristics of CD8^+ ^T cells from the PBMCs and TILs of individual NPC patients (Table 1), and we included matched healthy individuals as controls. We examined the percentage of CD8^+^Foxp3^+ ^Tcregs from PBMCs and freshly isolated TILs of NPC patients by analyzing the expression of CD8 and Foxp3 (Figure 1). The proportion of CD8^+^Foxp3^+ ^Tcregs was increased significantly in PBMCs from NPC patients (mean = 3.13 ± 5.04%, *n *= 21) as compared with healthy donors (mean = 0.8 ± 1.03%, *n *= 21; *P *< 0.05, Figure 2A), and the proportion of Tcregs in TILs (mean = 2.67 ± 3.36%, *n *= 21) was similar to that in PBMCs from paired NPC patients (Figure 2B). In addition to the Tcregs, the CD8^+ ^T cells contained a population of Tc17 cells, and we found that the percentage of Tc17 cells in PBMCs from NPC patients (mean = 0.29 ± 0.31%, *n *= 21) was significantly lower than that in PBMCs from healthy donors (mean = 1.04 ± 0.72%, *n *= 21) or TILs from the paired NPC patients (mean = 0.96 ± 1.12%, *n *= 21) (*P *< 0.005, Figure 2C, D)

**Table 1:** Sample information

**No**.	Gender	Age (yr)	Histological type^a ^	WHO classification^b^	Clinical stage
NPC1	F	52	III	T3N1M0	III
NPC2	M	29	III	T2N2M0	III
NPC3	M	53	III	T3N0M0	III
NPC4	M	45	III	T4N2M0	IVA
NPC5	M	46	III	T4N1M0	IVA
NPC6	M	47	III	T2N0M0	II
NPC7	M	63	III	T4N2M0	IVA
NPC8	M	36	III	T3N1M0	III
NPC9	M	44	III	T3N1M0	III
NPC10	F	29	III	T2N2M0	III
NPC11	M	38	III	T2N2M0	III
NPC12	F	31	III	T3N2M0	III
NPC13	M	39	III	T3N2M0	III
NPC14	F	25	III	T3N1M0	III
NPC15	M	38	III	T3N2M0	III
NPC16	M	34	III	T3N1M0	III
NPC17	M	38	III	T3N2M0	III
NPC18	M	42	III	T3N1M0	III
NPC19	M	30	III	T3N2M1	IVA
NPC20	M	54	III	T2N1M0	II
NPC21	M	56	III	T2N2M0	IVA

^a^Histological type: I = differentiated keratinizing squamous cell carcinoma; II = differentiated nonkeratinizing carcinoma; III = undifferentiated carcinoma. ^b^TNM stage according to WHO UICC-AJCC 1997 standards (International Union Against Cancer/American Joint Committee on Cancer)

### Phenotypic and functional features of the CD8^+ ^Tcreg and Tc17 subsets in NPC

**Figure 3:**
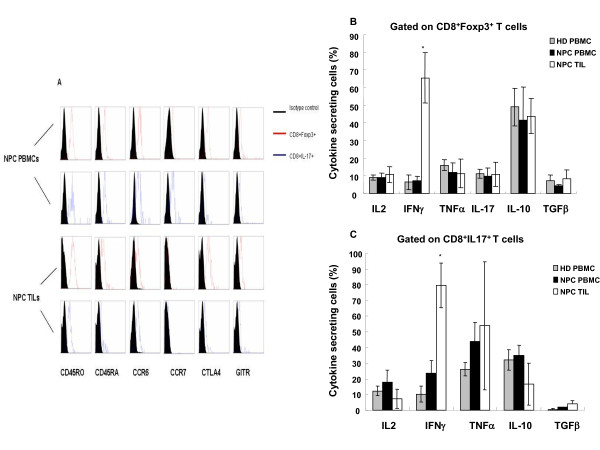
**Characterization of Tcregs and Tc17 cells in NPC**. (A) Expression of phenotypic markers, including CD45RO, CD45RA, CCR6, CCR7, CTLA4, and GITR, on Tcregs and Tc17 cells from PBMCs and TILs of one representative NPC patient. (B and C) Cytokine profiles of Tcregs (B) and Tc17 cells (C). Tcregs expressed high levels of IL-10 but low levels of IL-2, TNFα, IL-17, and IFNγ, except Tcregs from TILs, which expressed significantly higher levels of IFNγ compared with NPC PBMCs. Tc17 cells expressed high levels of TNFα, an intermediate level of IL-10, and low levels of IL-2 and IFNγ, except for cells from TILs, which also expressed significantly higher levels of IFNγ compared with NPC PBMCs. Data are from five patients with NPC and five healthy donors. *P < 0.005.

To characterize the subsets of Tcregs and Tc17 cells, we analyzed the phenotypic features of these cells (Figure 3A). The Tcregs in both PBMCs and TILs expressed CD45RO and CD45RA, indicating that this subset contained memory Tcregs and naïve Tcregs. The Tc17 cells from PBMCs also expressed CD45RO and CD45RA; however, the Tc17 cells from TILs were positive for CD45RO protein but negative for CD45RA. This indicates that Tc17 cells contained memory and naïve Tc17 cells in PBMCs but only memory Tc17 cells in TILs. Moreover, the Tcregs and Tc17 cells in PBMCs showed high levels of CCR6 and CCR7, whereas these subsets in TILs expressed high levels of CCR6 and low levels of CCR7. The Tcregs also expressed specific molecular markers of CD4^+ ^Tregs, including CTLA4 and GITR, whereas the Tc17 cells expressed moderate levels of CTLA4 but were negative for GITR protein.

Next, we evaluated the cytokine expression profile of Tcregs and Tc17 cells in PBMCs and TILs from NPC patients after stimulation with PMA/ionomycin *in vitro*. Data were acquired for PBMCs and TILs, and the assay was repeated 5 times with different donors, as shown in Figure 3B and 3C. Tcregs in PBMCs from NPC patients and healthy donors showed high expression of IL-10 and low expression of TGFβ, IL-2, IFNγ, TNFα, and IL-17. The Tcreg subset in TILs contained a significantly higher proportion of IFNγ-secreting cells than Tcregs from PBMCs of NPC patients and healthy donors (*P *= 0.001). The cytokine expression profile of Tcregs from TILs was IFNγ^high^IL-10^high^TGFβ^low^IL-2^low^TNFα^low^IL-17^low^, which indicated that Tcregs displayed a Tr1-like cytokine expression profile in the tumor microenvironment. The Tc17 cells also contained a high proportion of TNFα-secreting cells and some IL-10- and IL-2-secreting cells. Additionally, the Tc17 cells from TILs contained a much higher percentage of IFNγ-secreting cells than those from PBMCs of NPC patients or healthy donors (*P *< 0.0001).

**Figure 4:**
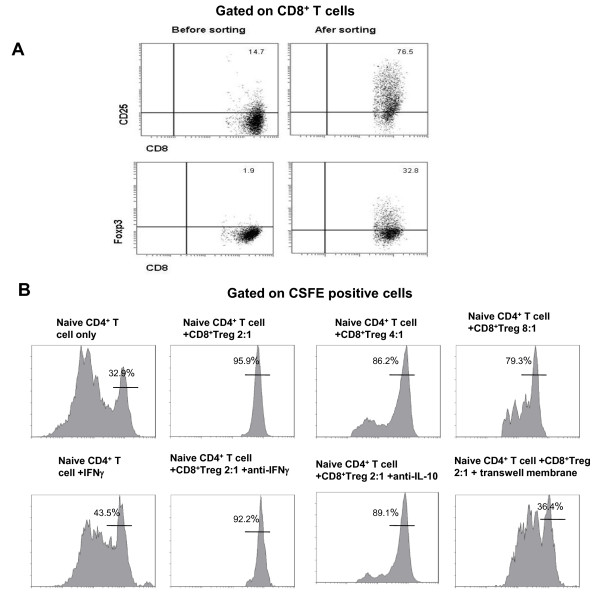
**Suppressive function of CD8^+ ^Tregs *in vitro***. (A) CD8^+ ^Tregs were isolated from TILs of NPC patients by sorting the CD8^+^CD25^+ ^T cells by flow cytometry. Representative dot plots for CD8, CD25, and Foxp3 before and after sorting are shown. (B) The suppressive activity of CD8^+ ^Tregs on naïve CD4^+ ^T cells *in vitro *was detected by proliferation of CSFE-labeled naïve CD4^+ ^T cells. CSFE-labeled naïve CD4^+ ^T cells were cultured alone or with CD8^+ ^Tregs at different ratios, in the outer and inner chamber of a transwell system, or in the presence of anti-IL-10 or anti-IFNγ antibodies in low IL-2 medium in 96-well or 24-well plates coated with OKT3 for 5 days. Representative results from one of five experiments are shown.

We investigated the suppressive function of Tcregs on naïve T cells *in vitro *by sorting CD8^+^CD25^+ ^T cells from peripheral PBMCs and TILs from NPC patients. The sorted CD8^+^CD25^+ ^T cells expressed a higher level of Foxp3 protein than the unsorted cells (Figure 4A), and they suppressed the proliferation of naïve CD4^+ ^T cells and CD8^+ ^effector T cells when the cells were co-cultured at different ratios *in vitro*, as do CD4^+ ^Tregs (Figures 4B and Additional file 1). This suppression could be recovered in a transwell system and was slightly inhibited by anti-IL-10 antibody but not by anti-IFNγ antibody (Figure 4B). This indicates that suppression by Tcregs *in vitro *was mainly mediated by cell-to-cell contact. Moreover, IFNγ also did not influence the suppressive function of Tcregs *in vitro*.

### EBV-antigen-specific CD8^+^Foxp3^+ ^Tregs were identified in NPC

**Figure 5:**
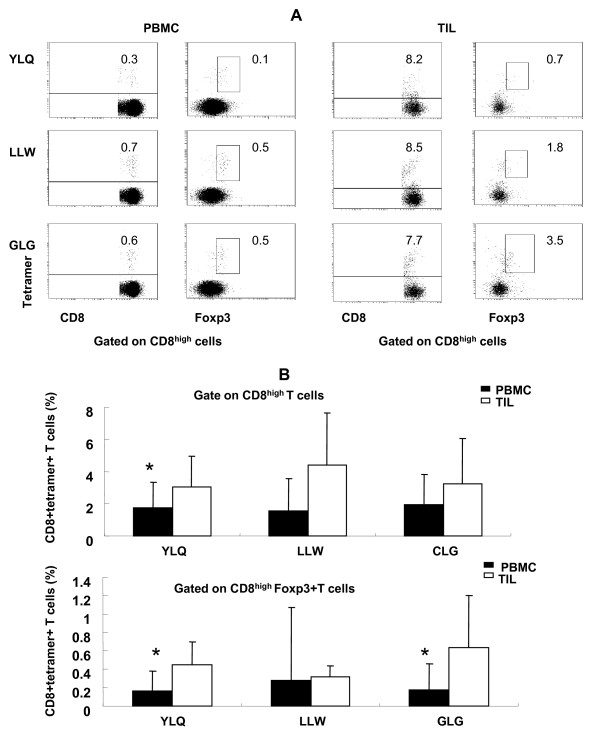
**Identification of EBV LMP1 and LMP2 epitope-specific CD8^+ ^T cells and Tcregs in NPC**. (A) CD8^+ ^T cells and Tcregs specific for the HLA-A2-restricted EBV epitopes LLW and YLQ (LMP1) and GLG (LMP2) in PBMCs and TILs were evaluated by FACS after staining for CD8, Foxp3, and tetramer. The results shown are from one patient. (B) The frequency of CD8^+ ^tetramer^+ ^T cells and CD8^+ ^Foxp3^+ ^tetramer^+ ^T cells in PBMCs and TILs. The results shown represent the mean ± SE of 12 PBMCs and 6 TILs from HLA-A2-positive patients with NPC. *P < 0.05 compared with TILs.

NPC tumor cells can present EBV latent type II antigens, including LMP1, LMP2, EBNA1, and BARF0. EBV-antigen-specific CD8^+ ^effector cells have been identified in EBV-associated malignancies [18]. However, it remains unclear whether EBV antigens activate pathogenic Tcregs in NPC. To investigate the existence of EBV-antigen-specific Tcregs in NPC, we used tetramer staining to determine the frequency of CD8^+ ^T cells and Tcregs from PBMCs and TILs of HLA-A2-positive NPC patients that were specific for the HLA-A2-restricted EBV epitopes LMP1 (YLQ) and LMP2 (LLW, GLG). From the CD8^high ^cell population, LMP1 (YLQ) and LMP2 (LLW, GLG) antigen-specific CD8^+ ^T cells and CD8^+^Foxp3^+ ^Tregs (Tcregs) were isolated from PBMCs and TILs of NPC patients (Figure 5A); however, only antigen-specific CD8^+ ^T cells but not CD8^+^Foxp3^+ ^Tregs (Tcregs) were observed in PBMCs of HLA-A2 positive healthy donors (Additional file 2). Notably, the frequency of LMP2 epitope LLW antigen-specific CD8^+ ^T cells was significantly higher in TILs than in PBMCs, as was the frequency of YLQ (LMP1) and GLG (LMP2) antigen-specific Tcregs (Figure 5B) (P < 0.05). This indicates that the EBV antigen-specific T effector cells and Tcregs can home to or be induced in NPC tumor tissues.

## Discussion

The development and progression of cancer are associated strongly with the unique immunosuppressive characteristics of the tumor microenvironment, regardless of the chronic inflammatory response and infiltrating inflammatory cells that are often produced around tumor tissue [19-22]. The increase and accumulation of CD4^+ ^Tregs in circulating PBMCs and tumor tissues have been observed in many kinds of malignancy, including NPC [23-26]. In a recent study, we showed by double immunohistochemical staining that there was a CD8^+^Foxp3^+ ^TIL subset in NPC tissues *in vivo *[17].

In the present study, we showed that the percentage of CD8^+ ^Tcregs increased in both circulating PBMCs and TILs of NPC patients when compared with PBMCs from healthy individuals (Figure 1, Figure 2A and 2B). This indicates that the accumulation of Tcregs in tumor tissue not only results from their recruitment from the peripheral blood to tumor tissue but also from the induction of CD8^+ ^Tcreg precursor cells at the tumor site. As a consequence, this Tcreg subset should contain natural Tregs (nTregs) and induced Tregs (iTregs) together at tumor site. This hypothesis was supported by the following findings. First, the Tcregs in TILs expressed CD45RO and CD45RA, which implied that Tcregs contained both memory and naïve Tcreg cells. Second, the Tcregs in PBMCs showed high levels of expression of CCR6, a ligand of chemokine CCL20, which is usually overexpressed in the NPC tumor microenvironment and upregulated by the EBV-encoded LMP1 protein [27,28]. Thus, these cells had migrated to the tumor tissue. Third, the Tcregs from the TILs expressed an IFNγ^high^IL-10^high^TGFβ^low^IL-2^low^TNFα^low^IL-17^low ^cytokine secretion phenotype (Tr1-like cytokine profile) (Figure 3). Moreover, we found that the Tcreg subset also contained some IL-17-producing cells (approximately 10%) and that the Tcregs from TILs expressed a significantly higher level of IFNγ than those from PBMCs of patients and controls. These results indicate that Tcregs in TILs of patients with NPC were not stable and did secrete some proinflammatory cytokines, such as IL-17 and IFNγ.

Our data demonstrate that Tcregs from TILs of NPC patients can suppress the proliferation of CD4^+ ^naïve T cells and CD8^+ ^effector T cells *in vitro*. This suppression was mainly mediated by cell-to-cell contact, and IFNγ did not affect the suppressive function of Tcregs in NPC (Figure 4). The results are supported by others' reports on the mechanism of suppression by Tcregs [29-32], and they reveal unique functions for Tcregs in NPC tumor tissue. On one hand, Tcregs facilitate tumor growth by suppressing activated T cells; on the other hand, they secrete a large amount of the Th1 cytokine IFNγ, which inhibits tumor cell growth and promotes the differentiation of Th1 cells.

In recent years, attention has focused on the role of CD4^+ ^Th17 cells in solid tumors, but their function remains largely unclear. Some researchers have suggested that Th17 cells promote tumor growth through an IL-6/STAT3 pathway, up-regulation of IL-8, and induction of tumor angiogenesis [33-35]. Others believe that Th17 cells have an antitumor function and that the number of Th17 cells is associated with a better outcome for cancer patients. Additionally, Th17 cells amplified adoptive CTL immunotherapy in one mouse model [34,35], and we have identified one CD4^+ ^Th17 clone from an NPC patient that could inhibit melanoma growth in a NOD/SCID mouse model (unpublished data). However, the role of Th17 cells in NPC remains poorly understood.

In sum, the present study is the first to investigate the distribution, generation, and function of Tc17 cells in NPC. We found that the proportion of Tc17 cells was reduced significantly in PBMCs when compared to TILs from NPC patients or to PBMCs from healthy donors (Figure 2C and 2D). The Tc17 cells in TILs expressed only CD45RO and not CD45RA (Figure 3A), indicating that this cell population was derived from memory cells and migrated from the peripheral blood. In addition, the high levels of expression of CCR6 in the Tc17 cells from PBMCs also indicate that these cells were able to migrate to tumor tissue. Therefore, the proportion of Tc17 cells from PBMCs was reduced compared to TILs from paired NPC patients or PBMCs from healthy individuals.

Tc17 cells expressed high levels of IL-17 and TNFα, intermediate levels of IL-10, and low levels of IL-2. Similar to Tcregs, Tc17 cells in the TILs but not in the PBMCs of patients with NPC expressed high levels of IFNγ (Figure 3C). This was consistent with a recent report of a similar cytokine profile for Tc17 cells in human liver cancer [36]. Furthermore, other research groups have shown that Tc17 cells, after polarization *in vitro*, can inhibit the growth of B16 tumors in mouse models by secreting IFNγ *in vivo *[14,37]. Most Tc17 cells are derived from CD8^+ ^T cells that lacked the expression of T-bet and Eomes; however, the expression of T-bet and Eomes is important for IFNγ production and for the antitumor function of CD8^+ ^T cells, especially in TILs [38]. We also found that in NPC patients, only Tc17 cells from TILs expressed high levels of IFNγ, which implied that Tc17 from TILs have a Th1 cell function and might have an antitumor function.

To address whether EBV antigens could induce Tcregs in NPC, we analyzed Tcregs specific for the EBV HLA-A2-restricted LMP1 and LMP2 epitopes by tetramer and Foxp3 staining. We identified CD8^+ ^T cells and Tcregs that were specific for the LMP1 epitopes LLW and YLQ and the LMP2 epitope GLG both in the PBMCs and TILs from NPC patients (Figure 5). The percentage of LLW-, YLQ-, and GLG-specific CD8^+ ^T cells and Tcregs was increased slightly in TILs as compared to PBMCs, but this increase was only significant for the LLW epitope antigen-specific CD8^+ ^T cells and the YLQ and GLG epitope antigen-specific Tcregs. These findings demonstrate that EBV epitope-specific CD8^+ ^T cells and Tcregs could home to or be induced in NPC tumor tissues, and some EBV epitope antigens, specifically YLQ and GLG, induced more antigen-specific Tcregs in tumor tissues than others. CD4^+ ^Tregs that are specific for the LAGE1 and ARTC1 tumor antigens have been identified in melanoma, and EBNA1 P_561-573 _and P_607-619 _peptide-specific CD4^+ ^Tregs have been identified in PBMCs from healthy donors [39,40]. These reports indicate that some antigens activate Tregs preferentially. In sum, the present study is the first to identify EBV epitopes that can induce antigen-specific CD8^+ ^Tcregs in PBMCs and TILs of NPC patients. Our results suggest that when EBV antigen- or peptide-specific CTL immunotherapy is established for NPC patients, antigen selection may affect clinical efficacy.

## Conclusions

In summary, we determined for the first time that there were different subsets of CD8^+ ^T cells, including Tcregs and Tc17 cells, among the circulating lymphocytes and TILs of patients with NPC. The results of our study suggest that Tcregs have diverse functions, and they also indicate a possible antitumor function for Tc17 cells in NPC tissues. Moreover, we revealed for the first time the presence of EBV antigen-specific Tcregs in NPC. Taken together, all these findings provide new insights into the composition and function of CD8^+ ^T cells and will be helpful for the development of T-cell-based adoptive immunotherapy for NPC.

## Abbreviations

NPC: Nasopharyngeal Carcinoma; Tcregs CD8^+ ^regulatory T cells: Tc17 IL-17-producing CD8^+ ^T cells; Foxp3: Forkhead box P3; TILs: Tumor-infiltrating lymphocytes; PBMCs: Peripheral blood mononuclear cells; CTLA4: Cytotoxic T lymphocyte antigen 4; GITR: Glucocorticoid-induced tumor necrosis factor (TNF)-related; Tr-1: Cells Regulatory type 1 cells.

## Competing interests

The authors declare that they have no competing interests.

## Authors' contributions

Conceived and designed the experiments: JL, YXZ. Performed the experiments: JL, ZFH, GX, JH. Analyzed the data: JL, HZF, LMZ, CNQ. Contributed reagents/materials/analysis tools: HYM, FQ, HQM, QYC. Wrote the manuscript: JL, CNQ. All authors read and approved the final manuscript.

## Supplementary Material

Additional file 1 **Analysis of the suppressive function of Tcregs to the proliferation of CD8^+ ^effector T cells at different ratios**. Click here for file

Additional file 2 **Tetramer staining of EBV LMP1 and LMP2 epitope-specific CD8^+ ^T cells and Tcregs from PBMCs of healthy donors**. Click here for file
