# Supplementary figures and images for: Distribution, characterization, and induction of CD8+ regulatory T cells and IL-17-producing CD8+ T cells in nasopharyngeal carcinoma
Source: J Transl Med. 2011 Nov 4;9:189. doi: 10.1186/1479-5876-9-189 (PMC3223152; doi:10.1186/1479-5876-9-189)

## Slide 1
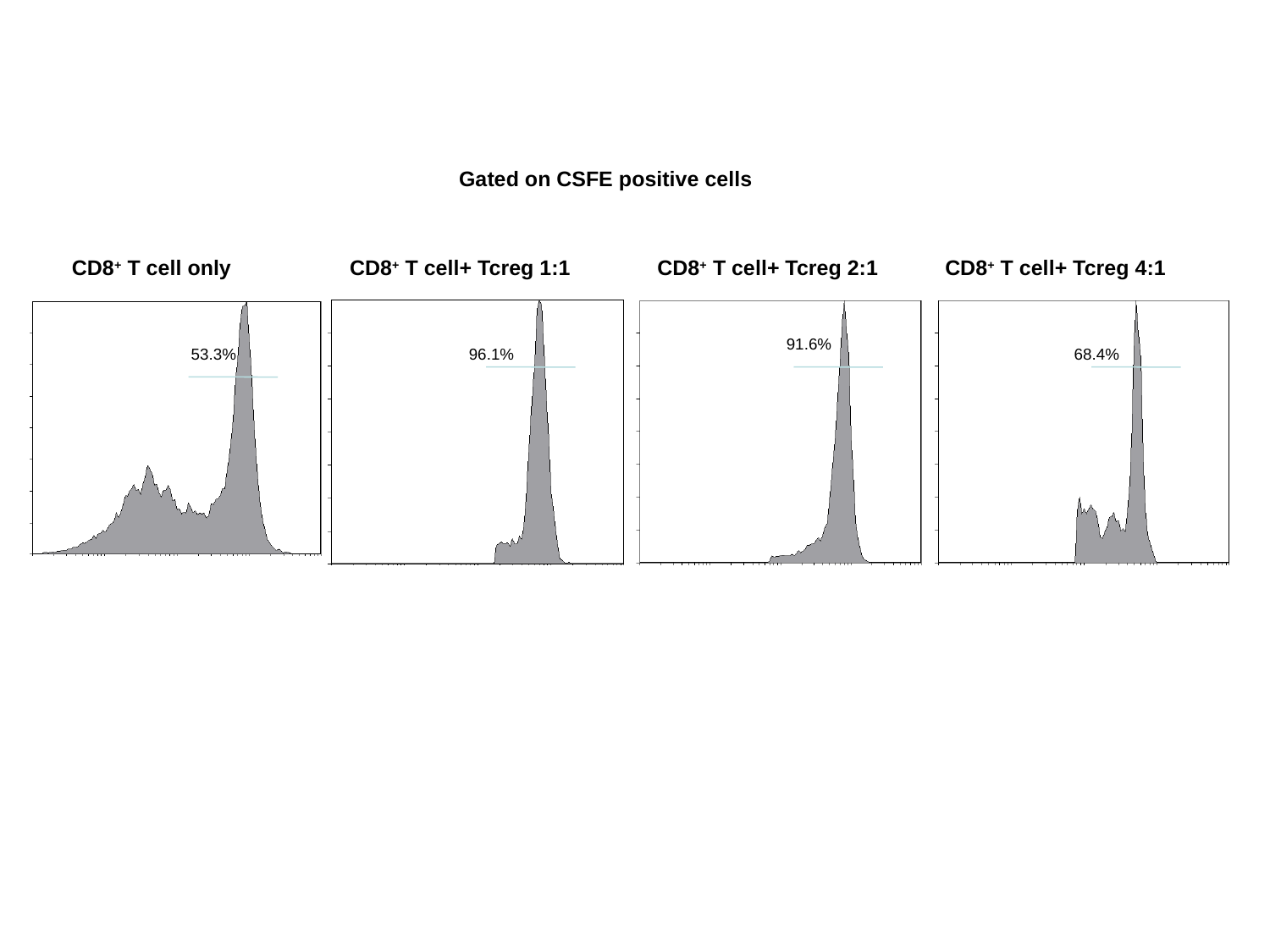

Gated on CSFE positive cells
CD8+ T cell only
CD8+ T cell+ Tcreg 1:1
CD8+ T cell+ Tcreg 2:1
CD8+ T cell+ Tcreg 4:1
91.6%
53.3%
96.1%
68.4%

Supplement: Additional file 1 — Analysis of the suppressive function of Tcregs to the proliferation of CD8+ effector T cells at different ratios. [file 1479-5876-9-189-S1.PPT]

## Slide 1
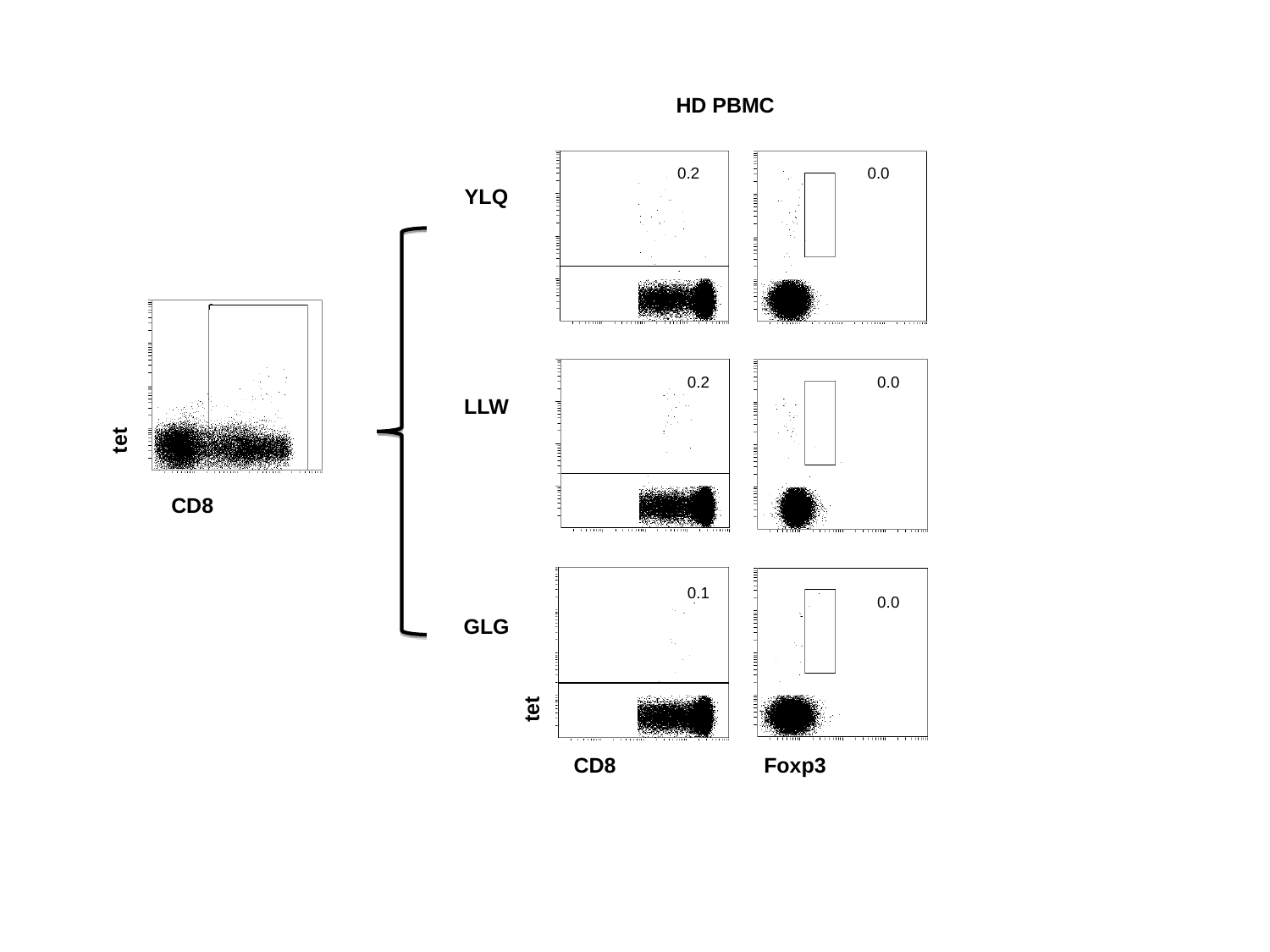

HD PBMC
0.2
0.0
YLQ
0.2
0.0
LLW
tet
CD8
0.1
0.0
GLG
tet
CD8
Foxp3

Supplement: Additional file 2 — Tetramer staining of EBV LMP1 and LMP2 epitope-specific CD8+ T cells and Tcregs from PBMCs of healthy donors. [file 1479-5876-9-189-S2.PPT]
